# Metabolic Bone Disease in preterm newborn: an update on nutritional issues

**DOI:** 10.1186/1824-7288-35-20

**Published:** 2009-07-14

**Authors:** Valentina Bozzetti, Paolo Tagliabue

**Affiliations:** 1 U.O. Neonatologia e Terapia Intensiva Neonatale, Istituto Maria Letizia Verga, Ospedale San Gerardo, Monza, Italy

## Abstract

Osteopenia, a condition characterised by a reduction in bone mineral content, is a common disease of preterm babies between the tenth and sixteenth week of life. Prematurely born infants are deprived of the intrauterine supply of minerals affecting bone mineralization.

The aetiology is multifactorial: inadequate nutrients intake (calcium, phosphorus and vitamin D), a prolonged period of total parenteral nutrition, immobilisation and the intake of some drugs.

The diagnosis of metabolic bone disease is done by biochemical analysis: low serum levels of phosphorus and high levels of alkaline phosphatase are suggestive of metabolic bone disease. The disease can remain clinically silent or presents with symptoms and signs of rachitism depending on the severity of bone demineralisation.

An early nutritional intervention can reduce both the prevalence and the severity of osteopenia.

This article reviews the pathophysiology of foetal and neonatal bone metabolism, focuses on the nutrient requirements of premature babies and on the ways to early detect and treat osteopenia.

## Background

The continuous advances in intensive care of preterm newborns have led to a progressive decline of mortality in Institutions where facilities and expertise for respiratory resuscitation and respiratory distress syndrome are available. Infant mortality dropped among all races between 1980 and 2000. The survival rate depends on the gestational age of the newborn; actually the survival rates for very low birth weight (VLBW) are the following: for those weighing 501 – 750 g is 56% and for the ones above 750 is 88% [1]. However, the success in the survival achieved through an aggressive intensive care is not always paralleled by a subsequent fully healthy development of the newborn.

Among the common conditions of morbidity due to the prematurity (cerebral impairment, bronchopulmonary dysplasia, growth failure, retinopathy...) a growing interest is focusing now on the metabolic bone disease of the prematurity (MBD), also called osteopenia of prematurity.

This condition is characterised by a reduction in bone mineral content (osteopenia), with or without rachitic changes, and is caused by several nutritional and biomechanical factors.

An inadequate supply of nutrients (vitamin D, calcium and phosphorus), a prolonged period of total parenteral nutrition, immobilisation and the intake of some drugs are the main factors involved in the pathogenesis of osteopenia [2].

The MBD usually occurs between tenth and sixteenth week of life, but it may remain silent until severe demineralisation (a reduction of BMD of 20 – 40%) occurs.

The clinical picture is various, ranging from a totally silent condition to a clinical picture of overt rickets, with multiple fractures and other alterations, when the demineralisation is severe.

The purpose of this review is to focus on the recent advances in the understanding of the bone tissue metabolism and on the nutritional approach to prevent and to treat the MBD.

## Magnitude of the problem

The prevalence of MBD varies depending on gestational age, birthweight and kind of alimentation.

It occurs in up to 55% of babies born with weight under 1000 g [3] and 23% of infants weighing < 1500 g at birth [4] and it is especially frequent in babies under 28 weeks of gestation. The prevalence is 40% in premature infants who are breastfed, in contrast to 16% of those fed with a formula designed for preterm infants and supplemented with calcium and phosphorus [5,6].

Preterm infants with a complicated medical course and delayed nutrition are also at high risk for MBD. Actually in western countries there is a trend of decrease of gestational age and birthweight, so the frequency of the MBD is expected to further increase.

## Homeostasis of calcium -phosphorus

The homeostasis of calcium, phosphorus and magnesium is fundamental for structural matrix of the bone.

Calcium and phosphate represent the major inorganic constituents of bone. The highest amount of calcium (99%) and of phosphorus (80%) of the whole body is in the bone as microcrystalline apatite.

Only 1% of the total body calcium is within the extracellular fluids and soft tissues. About the 50% of total serum calcium is in the ionised form and represents the biologically active part. A further 8–10% is bounded to organic and inorganic acid and the remaining percentage of calcium is protein-bound (80% to albumin, 20% to globulin).

The formation of the apatite takes place if calcium and phosphorus are simultaneously available in optimal proportions.

Also magnesium is part of the bone matrix and the 60% of total body magnesium is in the bone.

Calcium and phosphorus homeostasis is a function of hormones, vitamin D and dietary intake, and depends on the intestinal absorption, skeletal accretion and reabsorption, and urinary excretion. [7]

Parathyroid hormone (PTH) is synthesised and secreted from the parathyroid glands in response to a reduction of serum level of ionised calcium. PTH regulates mineral metabolism and skeletal homeostasis through its action on target cells in bone and kidneys. It stimulates the reabsorption of calcium and excretion of phosphorus in the kidney and bone reabsorption of calcium. PTH also is able to activate the synthesis of calcitriol via stimulation of renal 25 (OH) D3-1-alpha-hydroxylase activities.

In its active form, 1, 25(OH) 2 vitamin D, stimulates the renal reabsorption of calcium and phosphorus. The synthesis of calcitriol is inhibited by elevated serum levels of calcium and phosphorus.

The combined actions of PTH and calcitriol maintain the adequate concentration of calcium in the extracellular fluids.

Kidneys contribute to maintain homeostasis of calcium; urinary calcium is one third derived from diet and the remaining from body stores, mostly bone.

Diuretics, as furosemide, increase renal calcium excretion.

## Prenatal bone physiology

The amounts of minerals required for a correct accretion of the skeleton are widely different depending on the age of the babies.

The period of greater skeletal development is during the intrauterine life and specifically during the last trimester. The bone volume increases significantly with gestational age and the high net bone formation activity is mainly due to modelling, with a rapidly increasing trabecular thickness (the trabecular thickening rate being approximately 240 times faster in the foetus than in the children).

The mineralization process is determined by synthesis of the organic bone matrix by osteoblasts (osteoid) onto which calcium and phosphate salts are deposited. This process increases exponentially between 24 and 37 weeks of gestation, reaching the 80% of mineral accretion in the third trimester [8].

During gestation the developing fetus receives supplies of energy, protein and mineral for adequate growth (1.2 cm/week) and bone development.

At term the newborn skeleton has a high physical density (expressed as bone mass divided by bone volume).

The foetal accretion of calcium and phosphate during the last three months of gestation is about 20 g and 10 g respectively, which represents accretion rates of 100–120 mg/kg/day for calcium and 50–65 mg/kg/day for phosphate [9].

A very important role in skeletal accretion of the foetus is played by the placenta. In fact the transfer of calcium from the mother to the foetus through the placenta occurs via an active transport done by the calcium pump in the basal membrane [10]. There is a 1:4 maternal to foetal calcium gradient [11].

Moreover, the placenta is able to convert vitamin D to 1,25-dihydrocholecalciferol which is fundamental for transferring phosphate to the foetus [12].

The foetus is maintained hypercalcemic in a high calcitonin and estrogen environment which promotes the modelling/remodelling ratio in favour of modelling and thus increasing the endocortical bone [13].

As a result, infants born prematurely will be deprived of the intrauterine supply of calcium and phosphorus affecting bone mineralization.

It is well known that a chronic damage to the placenta may alter the phosphate transport; this explains why babies with intrauterine growth restriction may be osteopenic.

Demineralization is also observed in infants born from mother with chorioamniositis and placental infection [14].

Maternal dietary intake of calcium is a factor implied in foetal bone accretion. A supplement of calcium (2 g from before 22 weeks of gestation) to women with a low dietary calcium intake resulted in higher bone mineral content (BMC) of the total body in infants born at term [15].

## Post-natal bone physiology

After birth the physical density of term newborns bones decreases by 30% in the first 6 months of life [13]. This is mostly due to an enlargement of the marrow cavity size, which occurs faster than the increase in the cross-sectional area of the bone cortex [16]. In term infants these postnatal changes are not accompanied by an increase in bone fragility and occur because bone is exposed to different conditions before and after birth.

First, there are important changes of hormonal environment: the reduction of maternal estrogens [17] and a postnatal increase of PTH level mainly due to a reduction of the calcium supply by the placenta [18].

As the serum calcium levels falls in the first day of life, PTH secretion is stimulated. During this transition the response of the parathyroid gland to falling levels of ionised calcium is blunted, as emphasized in a recent review article [19]. This finally results in a physiological nadir in neonatal serum calcium levels within the first 48 hours of life. Of note, PTH level is still within the normal range for term babies or adult, but represents a decrease from foetal levels.

Many factors affect calcium absorption including the maternal vitamin D status, solubility and bioavailability of calcium salts, quality and quantity of calcium, amount and type of lipids and, obviously, gut function.

Calcium absorption from the intestine occurs both passively and through a vitamin-D dependent active transport mechanism. In a newly born preterm the low mineral content of human milk associated with a poorly efficient absorption of the developing gut determine a net reduction of calcium and phosphorus supply.

Absorption of phosphorus takes place in the jejunum and depends on the dietary intake. The phosphorus supply regulates calcium absorption and retention: the higher is the phosphorus content of the diet, the higher is the calcium retention. However, an excessive amount of one decreases the absorption of the other.

Moreover, while in utero fetus experiments mechanical stimulation by kicking against the uterine wall, this kind of training is missing during the extrauterine life since preterm babies usually stay in the incubator [20,21]. Inactivity due to immobilisation stimulates bone reabsorption by osteoclasts and urinary calcium excretion; furthermore the reduced muscle activity prevents the addition of new bone tissue [22]. Conditio sine qua non for the physical activity to be beneficial is that an adequate mineral intake is guaranteed [23].

**Figure 1:**
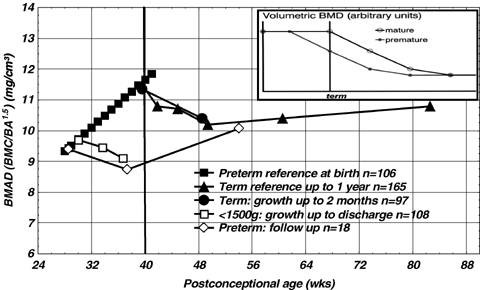
**Physiological evolution of DEXA apparent bone mineral density during the last trimester of gestation (*filled squares*) and during the first year of life in healthy term infants (*upper triangles*) compared to that observed in preterm infants (*open squares and lower triangles*)**. (With permission from Ref [12]).

The figure 1 shows that during the third trimester of gestation, bone mineral apparent density (BMAD) increases at a faster rate in utero (term infants) than ex utero (preterm infants) according to gestational age.

BMAD is an estimation of volumetric BMD (g/cm3) calculated as bone mineral content/bone area (BMC/BA). The figure 1 also shows that there is a sharp reduction in BMAD in neonatal age followed by a stabilization that lasts all the first year of life ("black triangles"). A similar event occurs in preterm babies: from birth to the term, mineral retention sharply diminishes comparing with the foetal life, while the skeletal growth remains high. This leads to a reduction of bone density ("white squares"). A catch up mineralization occurs after discharge of VLBW so BMC spontaneously improves ("white rhombs").

Among the other pathogenic factors, also problems related to inadequate supply of calcium to babies, which require parenteral nutrition and interference of several drugs, may contribute to determine preterm osteopenia with an increasing risk of bones fractures.

The drugs mostly implied in pathogenesis of MBD include steroids, methylxanthines and diuretics. They stimulate osteoclasts activation, decrease calcium absorption, reduce osteoblasts proliferation and increase calcium renal excretion and hence increase the risk of poor bone mineralization [24-26].

## Neonatal mineral requirements

The requirements of calcium and phosphorus are based on demands for matching intrauterine bone mineral accretion rates.

Supplying calcium and phosphorus in parenteral nutrition is a challenge because of limited solubility of these two minerals. Calcium and phosphorus's solubility in nutrition admixtures depends on temperature, type and concentration of aminoacid, glucose concentration, pH, type and concentration of calcium salts, and presence of lipid and so on...

In parenteral nutrition calcium is administered as inorganic salt and phosphorus may be administered as inorganic sodium and potassium phosphate or sodium-glucose phosphate or glycerolphosphate, which are quite soluble in water.

The addition of cystein to lower pH of the parenteral admixtures improves the solubility of calcium and phosphorus.

For all such reasons it is not possible to supply these minerals according to the physiologic requirements of the preterm to reach an adequate bone mineralization.

In the transition period, most of VLBW neonates receive full or partial parenteral nutrition with the goal to maintain normal levels of calcium and phosphorus. Hypocalcaemia, in fact, is a common event during the first days of life because of the sharp decrease of the calcium supply by the placenta and the delayed release of PTH due to the immature response of the parathyroid glands.

Parenteral administration of 50–75 mg of calcium/kg/day can prevent early neonatal hypocalcaemia in preterm infants.

Through the parenteral administration of calcium and phosphorus (40–70 mg/kg/day and of 25–45 mg/kg/day respectively) it is possible to achieve 60 – 70% of intrauterine mineralization [27]. The best calcium to phosphorus ratio for bone mineralization is 1.7:1 [28-30].

In preterm babies receiving parenteral nutrition only limited amounts of vitamin D are required since calcium is given by vein and there is no need of calcitriol to facilitate the intestinal uptake.

Moreover only the parent compound needs to be administered since the preterm infant is able to hydroxylate the inactive form to the active one since the 24th week of gestation. It is now generally accepted the daily recommended dose of vitamin D is 400 U.I./day [18].

For the transitional period, when infants are weaned from parenteral nutrition to the enteral one, the aim usually is to maintain an adequate serum level of calcium and phosphorus. However the serum level of calcium is not a good marker of adequacy of calcium intake since the level is maintained stable at the expense of the bone. Therefore the clinicians should be aware that a normal serum level of both calcium and phosphorus are not guarantee for an adequate whole body accretion as in intrauterine life.

The enteral administration of calcium is fraught with many problems as regards the calcium bioavailability. Vomiting, large gastric aspirates, constipation and abdominal distension are quite common in preterm babies and the gut absorption capacity is impaired due to the immaturity of the gastrointestinal mucosa.

Calcium absorption depends on vitamin D status, solubility of calcium salts, quality and quantity of lipid intake. Moreover, in preterm babies, vitamin D demands are influenced by body contents at birth which depends on the duration of gestation and maternal vitamin status.

Current estimates of requirements for calcium, phosphorus and vitamin D in growing premature infants vary among international sources of recommendations [31-34] (Table 1).

**Table 1:** Minerals and vitamin D recommended intakes in growing preterm infants.

**Requirements**	**ESPGAN **[32]	**LSRO **[31]	**Atkinson **[33]	**Rigo **[34]
	**1987**	**2002**	**2005**	**2007**
Calcium (mg/kg/day)	70–140	150–220	120–200	100–160

Phosphorus (mg/kg/day)	50–90	100–130	60–140	60–90

Vitamin D (I.U./day)	800–1600		200–1000	800–1000
(I.U./kg/day)		90–225	150–400	

The human milk content is inadequate for preterm requirements since the content of calcium and phosphorus in preterm human milk is 31 mg/100 kcal and 20 mg/100 kcal [18] while the Life Science Research Office [31] suggests, for premature formulas, a dose approximately 4–6 times higher (123 to 185 mg Ca/100 kcal and 80 to 110 mg P/100 kcal). Even when VLBW are fed at high feeding volumes (180–200 mL/Kg), assuming calcium and phosphorus absorption of 70% and 80% respectively, this would provide only one-third of the in utero level of absorbed calcium and phosphorus [6]. Formula milk is richer in calcium and phosphorus than human one, but bioavailability is quite different. In formula fed infants, calcium absorption is usually less than with human milk, ranging from 35 to 60% of the intake. Hence the human milk intake has to be promoted, but a fortification with mineral and protein fortifier is necessary to achieve adequate nutrient intake.

With the current human milk fortifiers, containing highly soluble calcium glycerolphosphate, calcium retention reaches a level of 90 mg/kg/day (88% of the overall intake).

However the new human milk fortifiers available in the market still do not allow intakes of calcium comparable with the values achieved during the last trimester of gestation (100–120 mg/kg/day) which are considered the target mineral accretion for preterm infants, nevertheless the use of multinutrient fortification of human milk for premature infants is currently recommended.

A Cochrane systematic review and metaanalysis of human milk fortifiers, which however included studies on children who were not extremely preterm (the class at major risk) stated that the effects on bone mineralization were not conclusive [35].

Finally, it must be noted that high calcium supplementation of milk is not well tolerated; it is associated with high faecal calcium, prolonged gastrointestinal transit time and impaired fat absorption. All these effects are potential risk factors for developing necrotizing enterocolitis.

(See table 1)

## Clinical features and diagnosis

MBD remains silent until a severe demineralisation occurs. The most evident clinical findings of osteopenia are deformity of the skull (diastasis of the suture, enlargement of the sagittal fontanelle and frontal bosses, craniotabe), thickening of the chondrocostal junctions and of the wrists, rib and long bones fractures. Softening and/or fractures of the ribs can cause pulmonary changes and respiratory distress, typically between 5 and 11 weeks of age [36].

Diagnosis of osteopenia is mainly done by serum analysis. Biochemically osteopenia is characterised by low serum levels of phosphorus and by an increase in serum levels of alkaline phosphatase that can reach values 5 times higher than the upper reference range used for adults [37]. It is useful dosing the isoenzimes of alkaline phosphatase since this enzyme is synthetised also by the liver and by the gut.

Backstrom and colleagues suggested that serum alkaline phosphatase levels higher than 900 U.I/l associated with a serum phosphate level lower than 1.8 mmol/l have a diagnostic sensitivity of 100% and specificity of 70% [38]. However the opinions in literature about the reliability of alkaline phosphatase to predict the status of bone mineralization are still conflicting [39,40]

Serum level of calcium is usually within the normal range due to effects of PTH on the bone. Low concentrations of calcium and phosphorus in the urine suggest an inadequate intake. This is manly due by an increase of the tubular reabsorption of phosphate because of the low dietary intake and by an increase of PTH level that stimulates the reabsorption of calcium. Markers of nutritional status should be assessed baseline, and then weekly during the initial phase; once the newborn is stable, assessment must be done at the starting of total enteral nutrition and successively every 2–3 weeks. If MBD is diagnosed and nutritional supplementation is started, a periodic assessment of laboratory data is necessary to evaluate the response to treatment also when babies are discharged from hospital. The key clinical goal is to maintain normocalcemia and normophosphatemia and to avoid an excessive calciuria.

Once levels of ALP, calcium and phosphorus normalize, serum analysis can be performed monthly up to 6 months of age and then every 3 months.

X-rays examination may show fractures, thin bones and other alterations as reduction of thickness of the cortical, enlargement of the epiphysis, irregular border between growth cartilage and bony metaphysis [41].

Dual energy X-ray absorbitometry (DEXA) is able to determine the bone mass content of neonates and can predict the risk of fractures [39,42] since it is sensitive in detecting small changes in BMC and BMD. Its use is now validated in neonates both term and preterm ones.

DEXA reflects most accurately the state of bone mineralization in preterm infants [43] but the examination involves radiations for the baby and the device is not portable.

Quantitative ultrasound is simpler than DEXA and is non-invasive; it can be used bedside without moving the baby. Reference values are now available for infants. Quantitative ultrasound gives information about structure of the bone and about bone density [44].

Osteopenia has a good prognosis since the disease is self-resolving, provided that calcium, phosphates and vitamin D are appropriately administered to the babies.

It is still controversial the need for high calcium and phosphorus intakes in preterm infants after hospital discharge. Few data are available about the optimal length, quantity and methods of providing supplemental minerals for preterm infants who are in stable growth.

There are studies that show increased bone mineral mass in infants who receive formulas containing more minerals that the traditional ones up to 9 months [45,46].

It has been shown, with studies assessing bone mineralization with quantitative ultrasound and DEXA, that preterm infants show a catch-up mineralization for the first year of life. There is no difference in late childhood of bone mineralization between term and ex-preterm infants [47] even though the biochemical evidence of metabolic bone disease during the neonatal period may have a long-term stunting effect which continues up to 12 years later. A recent study published on Journal of Perinatology [48] stated that children who were born prematurely with birth weights less than 1.5 kg tend to be significantly smaller for age and have lower lumbar spinal bone mineral content and density compared with children born at term gestation.

The long duration of this complication provides further rationale for implementing any practice that can prevent this condition [49].

In the case of BMD of prematurity nutrition is both therapy and prevention. An adequate intake of minerals and of vitamin D, with breast milk fortifier or formula with a content of minerals suitable for preterm infant's requirements, are necessary for a correct bone mineralization.

A regular physical stimulation, when the preterm infant is clinically stable and is receiving adequate doses of calcium, phosphate and vitamin D, should also be included in the standard preventive approach.

## Conclusion

An adequate nutritional intake of calcium, phosphorus and vitamin D and passive physical exercise may prevent abnormal bone-remodelling activity during first weeks of life and may optimize growth potential of preterm infants. It is important to recognize the biochemical signs of osteopenia in an early stage in order to be able to precociously implement the dietary intake and reduce the risk of bones fractures. The determination of alkaline phosphatase and of phosphoraemia seems to be useful in assessing the risk of metabolic bone disease and serum analysis need to be performed periodically in order to assess response to nutritional treatment. Through DEXA and quantitative ultrasound it is also possible to determine the state of bone mineralization and therefore to plan a nutritional intervention.

## Competing interests

The authors declare that they have no competing interests.

## Authors' contributions

VB and PT equally contributed at the article, analyzing the literature and writing the paper. All authors read and approved the final manuscript.
